# The Veterans Affairs Neuropathy Scale: A Reliable, Remote Polyneuropathy Exam

**DOI:** 10.3389/fneur.2019.01050

**Published:** 2019-11-01

**Authors:** Andrew M. Wilson, Michael K. Ong, Debra Saliba, Nasheed I. Jamal

**Affiliations:** ^1^Department of Neurology, VA Greater Los Angeles Healthcare System, Los Angeles, CA, United States; ^2^Department of Neurology, University of California, Los Angeles, Los Angeles, CA, United States; ^3^Department of Medicine, VA Greater Los Angeles Healthcare System, Los Angeles, CA, United States; ^4^Geriatric Research, Education, and Clinical Center, VA Greater Los Angeles Healthcare System, Los Angeles, CA, United States; ^5^Borun Center for Gerontological Research, University of California, Los Angeles, Los Angeles, CA, United States

**Keywords:** neuropathy, telemedicine, reliability, neuromuscular, examination

## Abstract

**Introduction:** Polyneuropathy (PN) complaints are common, prompting many referrals for neurologic evaluation. To improve access of PN care in distant community clinics, we developed a telemedicine service (patient-clinician interactions using real-time videoconference technology) for PN. The primary goal of this study was to construct a remote exam for PN that is feasible, reliable, and concordant with in-person assessments for use in our tele-PN clinics.

**Methods:** To construct the VA Neuropathy Scale (VANS), we searched the literature for existing, validated PN assessments. From these assessments, we selected a parsimonious set of exam elements based on literature-reported sensitivity and specificity of PN detection, with modifications as necessary for our teleneurology setting (i.e., a technician examination under the direction of a neurologist). We recruited 28 participants with varying degrees of PN to undergo VANS testing under 5 scenarios. The 5 scenarios differed by mode of VANS grading (in-person vs. telemedicine) and by the in-person examiner type (neurologist vs. technician) in telemedicine scenarios. We analyzed concordance between the VANS and a person's medical chart-derived PN status by modeling the receiver operating characteristic (ROC) curve. We analyzed reliability of the VANS by mixed effects regression and computing the intraclass correlation coefficient (ICC) of scores across the 5 scenarios.

**Results:** The VA Neuropathy Scale (VANS) tests balance, gait, reflexes, foot inspection, vibration, and pinprick. Possible scores range from 0 to 50 (worst). From the ROC curve, a cutoff of >2 points on the VANS sets the sensitivity and specificity of detecting PN at 98 and 91%, respectively. There is a small (1.3 points) but statistically significant difference in VANS scoring between in-person and telemedicine grading scenarios. For telemedicine grading scenarios, there is no difference in VANS scores between neurologist and technician examinations. The ICC is 0.89 across all scenarios.

**Discussion:** The VANS, informed by existing PN instruments, is a promising clinical assessment tool for diagnosing and monitoring the severity of PN in telemedicine settings. This pilot study indicates acceptable concordance and reliability of the VANS with in-person examinations.

## Introduction

Polyneuropathy (PN) complaints are common, prompting many referrals for neurologic evaluation. In our healthcare system, many of these referrals come from distant community clinics that do not have neurologists on site and serve a disproportionate number of adults for whom access to urban referral centers can be particularly difficult (1). To improve access and convenience of PN care for populations served in community clinics, we piloted a telemedicine service (patient-clinician interactions using real-time videoconference technology) for PN.

Telemedicine has expanded the ability of specialists to evaluate and treat chronic conditions remotely (2). Within the field of neurology, telemedicine (or teleneurology) has been employed successfully in chronic conditions principally assessed by history or visual inspection, such as dementia, epilepsy, and movement disorders (3, 4). However, teleneurology for neuromuscular disorders, such as PN, has not been trialed extensively, given the need for “hands-on” physical exam elements (e.g., eliciting reflexes, testing sensation) to assist with diagnosis and staging of the disease (5). Thus, the primary goal of this study was to develop a remote exam for PN that was feasible, reliable, and concordant with in-person assessments for use in our tele-PN clinics.

Our exam, the VA Neuropathy Scale (VANS), is a standardized PN assessment that resembles a focused-in person neuromuscular exam. The VANS was informed by existing, validated PN assessments and was modified for a clinical teleneurology setting (i.e., a technician examination under the direction of a neurologist). This article describes the development and pilot testing of the VANS for remote PN assessment and describes the VANS psychometric properties for this purpose.

## Methods

### Development of the VA Neuropathy Scale (VANS)

We searched the literature for clinical neuropathy exams using the PubMed search terms of (“polyneuropathy” OR “polyneuropathies” OR “neuropathy” OR “neuropathies”) AND (“scale” OR “score” OR “exam” OR “assessment”) AND (“sensitivity” OR “sensitive” OR “specificity” OR “specific” OR “reliability” OR “reliable” OR “validity” OR “valid”) in the title or abstract. The search resulted in 385 articles. We (AMW, NIJ) scanned the titles and abstracts to identify neuropathy exam scales. We excluded symptom checklists or exams requiring specialized quantitative tools. The search resulted in 10 PN assessments, which we deconstructed into discrete exam elements. We evaluated exam elements for inclusion into the VANS based on literature-reported sensitivity and specificity of PN detection as well as our team's judgement of exam element difficulty within the telemedicine setting. We assessed difficulty as challenges to the examiner's technique and/or to the neurologist's observation of the exam element. We selected a parsimonious set of exam elements, with modifications as necessary, to form the VA Neuropathy Scale (VANS).

### Concordance and Reliability Testing of the VA Neuropathy Scale (VANS)

This study was approved as a quality improvement activity by the Greater Los Angeles Veterans' Affairs Healthcare System (GLA VA) IRB. The need for written informed consent was waived by the IRB subcommittee that approved the study. Informed verbal consent was obtained from all participants.

Our primary outcome for this pilot was to determine the agreement of VANS scores via intraclass correlation coefficient (ICC) across 5 different scenarios (see Table 1). The scenarios differed on (1) whether the mode of VANS grading was in-person vs. remote (i.e., telemedicine), and (2) for remote grading scenarios, whether the in-person examiner was a board-certified neurologist vs. a telehealth care technician (TCT, a medical assistant or licensed vocational nurse without neurological exam experience).

**Table 1:** The 5 scenarios (A-E) for VANS reliability testing.

**Scenario**	**In-person examiner**	**In-person grader**	**Remote grader**
A	MD#1	MD#1	
B	MD#2	MD#2	
C	MD#1 or MD#2		TeleMD#3 or TeleMD#4
D	TCT#1		TeleMD#3 or TeleMD#4
E	TCT#2		TeleMD#3 or TeleMD#4

*The table describes each scenario by listing the in-person examiner, the in-person grader (when applicable), and the remote grader (when applicable). In scenario A and B, participants are examined and graded by an in-person neurologist, similar to a typical face-to-face encounter. In scenario C, participants are examined by one of the in-person neurologists, but the grading is done by one of the remote tele-neurologists. In scenarios D and E, participants are examined in-person by a telehealth care technician (TCT), but the grading is done by one of the remote tele-neurologists. MD = neurologist; TeleMD = tele-neurologist; TCT = telehealth care technician*.

Aiming to test the VANS across a spectrum of patients, we followed a purposive sampling technique to enroll Veterans from our neurology clinic with the following 3 PN disease categories based on their recent neurologic note in the medical record: no PN, PN without falls (PN-F), and PN with falls (PN+F). We defined no PN as an absence of the entire set of neuropathic symptoms, neuropathic signs, and neuropathic diagnosis. To be defined as PN, patients must have met the “Probable Clinical PN” criteria from the Toronto Expert Panel's definition (“a combination of symptoms *and* signs of distal sensorimotor polyneuropathy with any two or more of the following: neuropathic symptoms, decreased distal sensation, or unequivocally decreased or absent ankle reflexes”) (6, 7). We segmented the PN group into those with or without falls within the last 3 months as a crude indicator of functional severity.

We screened the medical charts of the 100 Veterans from the GLA VA region who had been referred to neurology for neuropathic complaints since January 2016 and who subsequently had an encounter diagnosis of PN from the neurologist. We also screened the charts of 20 Veterans without neuropathic complaints who had a recent neurology visit. Documentation was sufficient to place all 120 individuals into one of the 3 defined PN categories.

**Figure 1:**
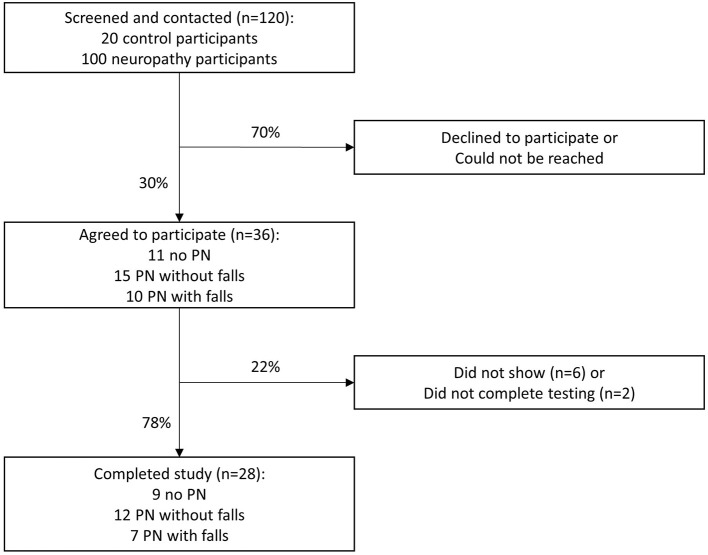
VANS study recruitment algorithm. PN, polyneuropathy.

With a desired sample size of 32 patients, our study was powered to detect a 95% confidence interval (CI) half-width of 0.15 from our hypothesized ICC of 0.7 across the 5 scenarios (8). We contacted all 120 Veterans in an attempt to enroll 40 participants, with at least 10 individuals in each of the 3 PN categories. The slightly larger enrollment was to protect from participant attrition. Ultimately, 36 Veterans agreed to participate. The recruitment algorithm for reliability testing of the VANS is illustrated in Figure 1.

After verbal informed consent was obtained from the 36 participants, they were asked to come to the West Los Angeles VA facility on a single day to have VANS testing under 5 scenarios (see Table 1). Twenty-eight of the 36 Veterans arrived for reliability testing. Four individuals completed only 4 exams rather than 5 exams due to scheduling conflicts.

We block randomized participants 1:1 to the two remote neurologists (TeleMD#3 or TeleMD#4) in each scenario of C, D, and E. For scenario C, we additionally block randomized participants 1:1 to the two in-person neurologists (MD#1 or MD#2). The order of testing for each patient was randomized to account for possible order effects in which participant behavior or provider performance might be influenced by prior exams. All neurologists and technicians were blinded to the patients' medical record-derived PN category. Schedules were arranged such that all participants could complete the 5 exams within 3 h. The 2 in-person neurologists and 2 technicians had received 15 min of basic exam technique training and 5 mock patient encounters (~30 min total) to learn the exam.

For analysis, we provide the descriptive statistics of the VANS scores for the cohort and by PN disease category (no PN, PN-F, PN+F). Regression analysis on clustered data was performed to demonstrate the relationship between PN category and VANS scores. We depict the non-parametric receiver operating characteristic (ROC) curve of the VANS and calculate the area under the curve (AUC) to illustrate the concordance between the medical record-derived binary PN category (no PN vs. PN with or without falls) and a VANS PN indicator. An AUC of 0.5 indicates chance agreement between the medical-record disease state and the VANS PN indicator while an AUC of 1 indicates perfect agreement. We empirically identify the “statistically best” cutoff for the VANS score to determine the presence of PN by both the Liu and Youden methods (9). Based on this cutoff, we calculated the sensitivity, specificity, positive predictive value, and negative predictive value of the VANS for this purposive sample.

For interrater reliability, we computed the absolute agreement, individual intraclass correlation coefficient (ICC) across the 5 scenarios using a mixed effects model. In the model, participants were the random effect with scenario as the fixed effect. After computing the mixed effects models, contrasts of fixed effects parameters were calculated to test differences in scores between our “gold standard” in-person examinations (scenarios A and B) and teleneurology examinations (scenarios C, D, and E). For intra-rater reliability, we calculated Spearman correlations and performed Wilcoxon signed-rank test for VANS scoring when either the in-person neurologist repeated the exam on the same person (scenario A or B with C) or the remote neurologist repeated the exam (scenario C with D or E OR scenario D with E). Analysis was performed using STATA v15.

## Results

### Development of the VA Neuropathy Scale (VANS)

The development of the VANS was informed by the following list of validated assessments that screen for and monitor PN: the Neuropathy Disability Score (NDS); the Michigan Neuropathy Screening Instrument (MNSI); the Michigan Diabetic Neuropathy Score (MDNS); the Total Neuropathy Score (TNS); the Neuropathy Impairment Score-Lower Limb (NIS-LL); the Diabetic Neuropathy Examination (DNE); the Toronto Classification Scoring System (TCSS); the Utah Early Neuropathy Score (UENS); the modified Toronto Classification Neuropathy Score (mTCNS); and the Early Neuropathy Score (ENS) (10–18). Table 2 summarizes each assessment's exam elements, most of which were common to multiple assessments. The number of exam elements per assessment ranged from 3 to 8. Briefer assessments (e.g., MNSI, ENS) generally coincided with a purpose of clinical use/screening, while more comprehensive assessments (e.g., MNDS, NIS-LL) were typically utilized in monitoring PN changes in clinical trials. This was most apparent in the domain of reflex and strength testing. Assessments did vary in the weighting of exam elements; some assessments assigned more points to severe distal deficits of particular exam elements (e.g., MDNS), while others assigned more points to the *spread* of deficits. The TCSS is an example of spread to different fiber types as it provides superficial coverage for distal motor, large-fiber, and small-fiber neuropathy. The mTCNS is an example of centripetal spread of sensory deficits to more proximal body regions. The mTCNS does not include reflex or strength testing at all. Unique to the UENS, pinprick sensation was tested over 6 leg regions, a feature that made it more sensitive to small-fiber neuropathy.

**Table 2:** Overview of existing polyneuropathy assessments.

**Item**	**Revised NDS**	***MNSI***	**MDNS**	**TNS**	**NIS-LL**	**DNE**	**TCSS**	**UENS**	**mTCNS**	**ENS**	**VANS**
Pin sensation	At toe: [0–1]		At toe: 0 = painful 2 = not painful	[0–4] region	At toe: [0–2]	At toe finger: [0–2]	At toe: [0–1]	[0–2] in each of 6 leg regions	[0–3] region	At toe: [0–2]	[0–2] in each of 6 leg 2 hand regions
Temperature	At toe: [0–1] using cold tuning fork						At toe: [0–1]		[0–3] region	At foot: [0–2] using cold thermal disks	
Position sense					At toe: [0–2]	At toe: [0–2]	At toe: [0–1]	At toe: [0–2]	[0–3] region		
Vibration	At toe: [0–1]	At toe: [0–2] 0.5	At toe: [0–2]	[0–4] region	At toe: [0–2] using 165 Hz fork	At toe: [0–2]	At toe: [0–1]	At toe: [0–2]	[0–3] region	At toe: [0–2]	At toe: [0–2] At knee: [0–2]
Light touch			At toe: [0–2] with SW monofilament		At toe: [0–2]	At toe: [0–2]	At toe: [0–1]		[0–3] region	At toe: [0–2] with SW monofilament	
Great toe extension			[0–3] severity	[0–4] severity	[0–4] severity			[0 or 2]			
Ankle reflexes	[0–2]	[0–2] *0.5	[0–2]	[0–2]	[0–2]	[0–2]	[0–2]	[0–2]		[0–2]	
Knee reflexes			[0–2]	[0–2], counted only if ankle reflexes absent	[0–2]		[0–2]				[0–1]
Other		*Foot visual: [0–1]*, additional point for ulcer	Strength—see note 1 Reflex—see note 2		Strength—see note 3	Strength—see note 4		Allodynia in toes/feet: [0–1]			Foot visual: [0–1] Romberg: [0–1] Gait - see note 5
References	Young et al. (10)	Feldman et al. (11)	Feldman et al. (11)	Chaudhry et al. (12)	Bril (13)	Meijer et al. (14)	Bril and Perkins (15)	Singleton et al. (16)	Bril et al. (17)	Zilliox et al. (18)	Present Study

*The table summarizes the PN exam items and scoring for each of the 10 PN assessments. The VA Neuropathy Scale (VANS) is listed in the last column for comparison. The 10 assessments are as follows: Revised Neuropathy Disability Score (NDS); Michigan Neuropathy Screening Instrument (MNSI); Michigan Diabetic Neuropathy Score (MDNS); Total Neuropathy Score (TNS); Neuropathy Impairment Score-Lower Limb (NIS-LL); Diabetic Neuropathy Examination (DNE); Toronto Classification Scoring System (TCSS); Utah Early Neuropathy Score (UENS); modified Toronto Classification Neuropathy Score (mTCNS); Early Neuropathy Score (ENS). SW, Semmes Weinstein monofilament*.

*Grading Legend*.

*[0 or 2]: 0 = normal, 2 = weak. [0–1]: 0 = normal, 1 = abnormal. [0–2]: 0 = normal, 1 = reduced, 2 = absent. [0–3] region: 0 = normal, 1 = reduced at toes, 2 = reduced to ankle, 3 = reduced above ankles. [0–3] severity: 0 = normal, 1 = mild to moderate weakness, 2 = severe weakness, 3 = no strength. [0–4] region: 0 = normal, 1 = reduced in fingers/toes, 2 = reduced to ankle/wrist, 3 = reduced to knee/elbow, 4 = reduced beyond knee/elbow. [0–4] severity: 0 = normal, 1 = mildly weak, 2 = moderately weak, 3 = severely weak, 4 = paralysis. Note 1: Finger spread and ankle dorsiflexion rated [0–3] severity. Note 2: Biceps and triceps reflexes rated [0–2]. Note 3: Strength rated [0–4] severity, including partial points. Muscles tested includes hip flexion, hip extension, knee flexion, knee extension, ankle dorsiflexion, ankle plantar flexion, toe extension, toe flexion. Note 4: Quadriceps and tibialis anterior tested with score 0 = normal, 1 = at least antigravity, 2 = weaker than antigravity. Note 5: Gait is tested by casual, heel, and tandem walk with score rated [0–1]*.

Table 3 provides further details about the common exam elements: the literature reported sensitivity and specificity of PN detection, frequency of use across the 10 PN assessments, and our team's anecdotal experience and clinical judgement of the telemedicine difficulty of these exam elements. From the work of Abraham et al. in a cohort of 312 patients with PN, the most sensitive exam elements for PN are ankle reflexes (74%), vibration (73%), and pinprick sensation (72%) (19). The most specific elements for PN are position sense at toes (98%), light touch at toes (96%), and knee reflexes (96%) (19). Though not universally included in each assessment, the most frequently tested exam elements were vibration (10/10), pin sensation (9/10), and ankle reflexes (9/10). Items such as temperature sensation, position sense, light touch, strength testing, gait, and feet inspection were also noted. From our experience, the exam elements that require examiner practice beyond a 20-min training session are strength testing and reflexes. Exam elements that are difficult to observe remotely, and to redirect if necessary, are position sense at toes and ankle reflexes.

**Table 3:** Summary of PN exam element sensitivity, specificity, and telemedicine difficulty.

**Item**	**Sensitivity**	**Specificity**	**Frequency used**	**Difficulty**	**Difficulty comment**	**Other comments**
Pin sensation	72%	91%	9/10	-		
Temperature	60%	89%	4/10	-		
Position sense	36%	98%	5/10	+	Observability	Modified to Romberg
Vibration	73%	77%	10/10	-		
Light touch	45%	96%	6/10	-		
Great toe extension	44%	?	4/10	+	Technique	Modified to heel walk
Ankle reflexes	74%	62%	9/10	++	Technique, observability	
Knee reflexes	52%	96%	4/10	+	Technique	
Foot inspection	?	?	1/10	-		
Gait	43%	?	0/10	-		Modified to casual and tandem gait

*The table describes the sensitivity and specificity of exam elements in Abraham el al.'s cohort of 312 PN patients (19). The fourth column indicates the frequency of an item's use out of the 10 PN assessments listed in Table 1. The fifth column is our team's assessment of the difficulty of exam element use in the telemedicine setting, with the reason for each “+” challenge listed in the sixth column. An element gets a “+” for either challenges related to mastery of the exam technique by the examiner and/or to the remote neurologist observing (and correcting if need be) the exam technique or response. The final column illustrates how we modified the VANS as possible to include desired elements. VANS, VA Neuropathy Scale*.

**Figure 2:**
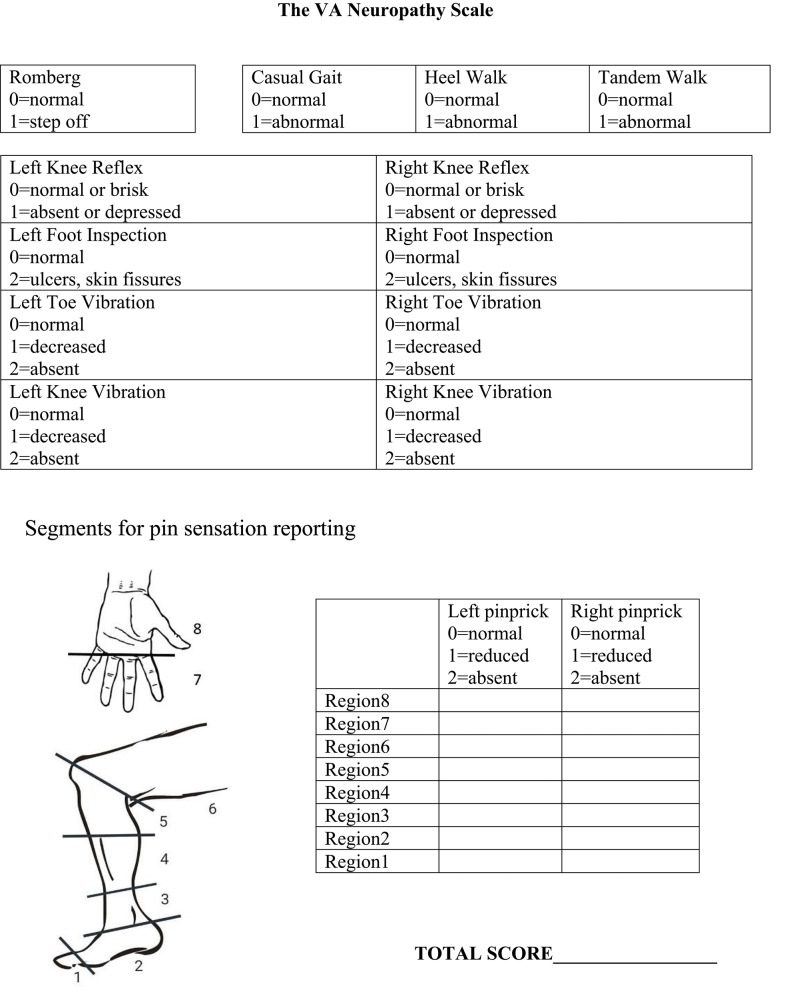
The VA neuropathy scale.

Based on a review of the above factors, we developed the VANS (Figure 2). We selected elements to optimize clinical utility, sensitivity and specificity of PN detection, monitoring of disease progression, and reliability. The VANS thus tests 6 exam elements: balance, gait, foot inspection, vibration, knee reflexes, and pinprick testing. The VANS scores can range from 0 (best) to 50 (worst) with examiners assigning points for abnormalities deemed attributable to distal symmetric PN. For balance, the Romberg test is performed with feet together, eyes closed for 20 s (20). Casual gait, walking on heels, and tandem gait (with feet touching in heel-to-toe orientation) are assessed with wide-based gait, steppage gait, or inability to perform the gait as abnormal. Knee reflexes are tested with a Queen Square hammer and are abnormal if depressed or absent. Inspection of feet is considered abnormal if there is skin breakdown, such as fissures or ulcers (dry skin, skin discoloration, or change in hair pattern does not count). Vibration using a 128 Hz Rydel-Seiffer tuning fork at the toe and knee is performed, with values 4 or below considered abnormal (21). If a patient does not feel any vibration on maximal stimulation, the assessor assigns 2 points. Pinprick sensation using Neurotips™ is performed by first demonstrating “normal” sharp on the forearm or, if necessary, the cheek. Participants can say whether the pinprick is “normal sharp,” “somewhat sharp,” or “not sharp at all” as the examiner moves up centripetally from the big toe or middle finger in roughly 2-cm increments until a level of change can be identified. Regions are scored based on the worst response for that region. The VANS scoring rubric is also seen on the rightmost column of Table 2 for ease of comparison to existing PN assessments.

### Concordance and Reliability Testing of the VA Neuropathy Scale (VANS)

Of the 28 participants, 24 (86%) were male and 15 (54%) were white. The average age was 64 years with a range of 26 to 85 years. The etiologies for PN in the 19 affected participants were as follows: diabetes (*n* = 5), pre-diabetes (*n* = 3), idiopathic (*n* = 3), alcohol abuse (*n* = 3), monoclonal gammopathy of unclear significance (*n* = 2), chemotherapy-induced (*n* = 2), and hereditary sensory motor neuropathy (*n* = 1).

**Figure 3:**
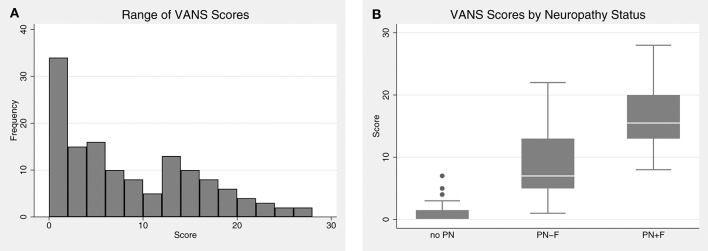
The distribution of VANS scores. **(A)** VANS scores for the entire cohort ranged from 0 to 28 with a median of 6. The mean was 8.1 with a standard deviation (SD) of 7.3. **(B)** There is a significant relationship between VANS score and PN status (*F*_2,27_ = 70.16; *P*-value < 0.0001). The average (SD) score of no PN, PN-F, and PN+F is 1.0 (1.5), 8.7 (5.3), and 16.5 (4.7) points, respectively. VANS, VA Neuropathy Scale; PN, polyneuropathy; PN-F, polyneuropathy without falls; PN+F, polyneuropathy with falls.

**Figure 4:**
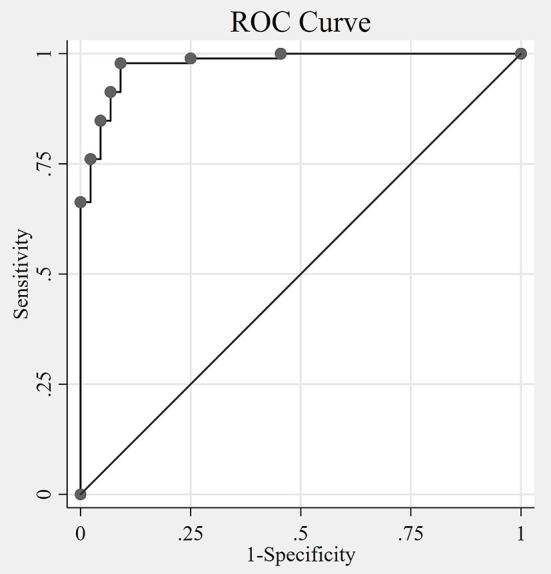
The Receiver Operating Characteristic (ROC) curve. The non-parametric ROC curve depicts the tradeoff of sensitivity and specificity of the VANS identifying polyneuropathy at different cutoff values of VANS scores. The area under the curve (AUC) is 0.98 (95% CI 0.95 to 0.99), with an AUC of 1 indicating perfect agreement between the VANS PN indicator and the actual disease status. The optimal cutoff by both Liu and Youden methods is 2 (meaning VANS scores >2 indicates the presence of PN). VANS, VA Neuropathy Scale; CI, confidence interval; PN, polyneuropathy.

VANS scores ranged from 0 to 28, with a mean of 8.1 and median of 6. Figure 3 shows the distribution of VANS scores for the cohort and by PN disease category. The average VANS scores by PN disease category were 0.95, 8.66, and 16.53 for no PN, PN-F, and PN+F, respectively. There was a significant association between VANS score and PN disease category (*F*_2, 27_ = 70.16; *P*-value < 0.0001). The ROC curve is shown in Figure 4 and has an area under the curve (AUC) of 0.98 (95% CI 0.95 to 0.99). The “statistically best” cutoff for defining PN by the Liu or Youden criteria was >2 points. The sensitivity and specificity of identifying PN with a score >2 was 98 and 91%, respectively. In this sample, the positive predictive value was 96%, and the negative predictive value was 95%. These results did not materially change when excluding those Veterans in the more impaired PN+F category (*n* = 7 participants).

**Figure 5:**
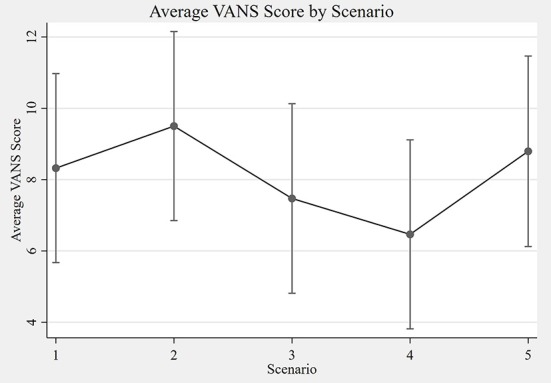
Average VANS score by scenario. The graph depicts the average VANS score by scenario with 95% confidence intervals. From left to right the mean VANS scores were as follows: A = 8.3; B = 9.5; C = 7.5; D = 6.5; E = 8.8. Please see Table 1 for a description of the scenarios A–E. VANS, VA Neuropathy Scale.

The overall ICC for the VANS across all scenarios was 0.89 (95% CI: 0.81 to 0.94). The ICC for only in-person encounters (A+B) was 0.91 (95% CI: 0.81 to 0.95). For telemedicine encounters (C+D+E) the ICC was 0.86 (95% CI: 0.75 to 0.92). The ICC for telemedicine encounters using telehealth care technicians (D+E) was 0.85 (95% CI: 0.71–0.93). Using a separate mixed effects model with both the in-person examiner and the remote grader as the fixed effects did not materially change the results. Table 4 summarizes the ICCs across the various scenarios. Figure 5 shows the average VANS score by scenario.

**Table 4:** Interrater reliability of the VANS.

**Scenarios**	**ICC (95% CI)**
Scenarios A–E: All scenarios	0.89 (0.81–0.94)
Scenarios A–B: Only in-person scenarios	0.91 (0.81–0.95)
Scenarios C–E: Only telemedicine scenarios	0.86 (0.75–0.92)
Scenarios D–E: Only TCT telemedicine scenarios	0.85 (0.71–0.93)

*Interrater reliability of the VANS is demonstrated by the intraclass correlation coefficients (ICCs) of the participants' VANS scores across different scenarios (see Table 1 for scenario descriptions). An ICC of 1 is perfect agreement in scores. The first column mentions the exam scenarios to be compared. The second column shows the ICC point estimates and 95% confidence interval for the specified scenario based on the mixed effects model. ICC, intraclass correlation coefficient; CI, confidence interval; VANS, VA Neuropathy Scale; TCT, telehealth care technician*.

Performing a contrast of in-person (A+B) vs. remote (C+D+E) VANS grading for the cohort shows in-person grading to have a statistically significant higher average score of 1.33 points (95% CI: 0.52 to 2.15). A similar difference of 1.28 (95% CI: 0.38 to 2.18) was found when comparing in-person (A+B) vs. remote with telehealth care technician exam (D+E) grading. A contrast of remote grading with an in-person neurologist (C) vs. telehealth care technician (D+E) shows no association between score and in-person provider type (0.16 lower with TCT; 95% CI: −1.27 to 0.95). Table 5 summarizes the grading score contrasts across scenarios, stratified by PN disease category. Of note, participants without PN showed no difference in score in any of the aforementioned comparisons. In addition, there was no difference in score in any PN disease category strata when comparing neurologist vs. telehealth care technician exams for remote grading scenarios.

**Table 5:** Comparison of VANS scores by scenario.

**Scenario comparisons**	**Polyneuropathy disease category**			
	**All**	**No PN**	**PN-F**	**PN+F**
In-Person vs. Remote	1.33^*^	0.17	1.90^*^	1.78
(A+B vs. C+D+E)	(0.52–2.15)	(−0.63–0.97)	(0.53–3.27)	(−0.05–3.61)
In-Person vs. TCT Remote	1.28^*^	0.22	1.71^*^	1.85
(A+B vs. D+E)	(0.38–2.18)	(−0.65–1.09)	(0.19–3.23)	(−0.18–3.87)
Neurologist vs. Technician (remote exams only)	−0.16	0.16	−0.58	0.20
(C vs. D+E)	(−1.27–0.95)	(−0.95–1.27)	(−2.43–1.26)	(−2.25–2.66)

*The table displays differences in scores (with 95% confidence intervals) based on post-regression contrasts of the mixed effects model. The first column describes the scenario groupings (see Table 1 for scenario descriptions). Column 2 displays the results for the entire cohort, while columns 3–5 display the results for the corresponding polyneuropathy disease category. Scores are positive when the first scenario grouping is higher than the second*.

* *P-value < 0.05; VANS, VA Neuropathy Scale; TCT, telehealth care technician; PN, polyneuropathy; PN-F, polyneuropathy without falls; PN+F, polyneuropathy with falls*.

In-person “intra-rater” exam agreement was Spearman rho = 0.95 (*n* = 27 participants). There was a statistically significant difference in score distribution between the (higher) in-person graded exam and the remote graded exam on the same participant with the same in-person neurologist (Wilcoxon *z* = 2.2, *P*-value = 0.03). Remote “intra-rater” exam agreement was Spearman rho = 0.90 (*n* = 28). There was not a statistically significant difference in score distribution between the neurology-presented exam and the TCT-presented exam on the same participant with the same remote tele-neurologist (Wilcoxon *z* = −1.1, *P*-value = 0.29).

## Discussion

### Development of the VA Neuropathy Scale (VANS)

We created the VA Neuropathy Scale (VANS) to help neurologists evaluate and manage PN via telemedicine. The VANS was our solution to the impracticality of training non-physician technicians to perform comprehensive neurological exams at each community site. For the tele-PN clinics in our VA region, the VANS has shown to be a promising clinical exam that is easy for technicians to learn and takes only 6 min to perform.

In addition, we felt that no existing PN assessment could be used in its original form for our telemedicine context. Previous papers have noted that PN assessments with lengthy scoring systems and sophisticated ancillary testing for a pre-specified disease etiology (e.g., inflammatory neuropathies, diabetic large-fiber neuropathy) limit their utility in most clinical contexts (18, 22) These limitations are amplified with telemedicine. Therefore, we modified exam elements from existing, validated assessments (summarized in Tables 2, 3) to improve telemedicine usability while still upholding the demonstrated strengths of the underlying assessments.

One theoretical strength of the VANS is that it detects subtle PN findings regardless of etiology. The VANS includes sensitive exam elements for both large-fiber (e.g., vibration) and small-fiber (e.g., pinprick) PN. While reduced ankle reflexes are the most sensitive marker of PN, their specificity and the ability of novice trainees to elicit reflexes accurately are low (19, 23). The mTCNS similarly excludes ankle reflexes and strength testing while maintaining the highest sensitivity of 7 PN scales in Zilliox et al.'s review (17, 18). In the place of ankle reflexes or confrontational strength testing, difficulty with heel walk is a common and early distal motor weakness sign in patients with PN due to weakness of the tibialis anterior. Another theoretical strength of the VANS is its potential to monitor disease progression by noting a change in severity (e.g., gradations of impairment) or spread (e.g., to more proximal regions or different fiber types) of sensory impairment. We adapted the pinprick testing from the UENS, which was a superior feature in detecting small-fiber neuropathy (16). The addition of knee reflexes and position sense with the Romberg heightens the VANS specificity (19).

Unlike other PN scoring systems, the VANS also incorporates tests for balance, mobility, and skin integrity to capture important vulnerabilities in more severe PN cases that warrant further intervention through physical therapy or podiatry. These common neurologic exam components can be performed easily in clinic, while providing indicators of functional status, similar to more structured tests such as the Dynamic Balance Test, the Functional Reach Test, and the Timed Up and Go Test (24).

### Concordance and Reliability Testing of the VA Neuropathy Scale (VANS)

The VANS is a promising scale that has good agreement with the medical record-derived, in-person evaluation of PN. Based on a cutoff of >2 points in this pilot study, the VANS has a high sensitivity (98%) and specificity (91%) for diagnosing PN. This result is on par with the validated instruments from which it was constructed (18). As we expected, average VANS scores increased across the PN disease categories: 0.95 for no PN, 8.66 for PN-F, and 16.53 for PN+F. While the primary outcome of this study was not to demarcate or declare disease severity, finding higher VANS scores in those with falls adds a modicum of criterion-related validity.

There was a statistically significant, but clinically equivocal, association between the score and mode of evaluation. Specifically, in-person exams had slightly higher scores (1.33 points) than remote exams. One plausible explanation is that remote exams may miss subtle PN signs, especially since the point difference was concentrated in the PN-F category. However, this difference did not impact the concordance of identifying the absence or presence of PN. The type of examiner in the remote scenarios did not impact the VANS score either.

Further, the VANS demonstrates excellent reliability, regardless of whether the exam is graded in-person or remotely, or whether it is performed by a neurologist or a telehealth care technician (TCT). The agreement among telemedicine encounters (ICC = 0.86) is nearly as high as the agreement among our gold standard in-person neurology encounters (ICC = 0.91). Interrater reliability (ICC = 0.89 across all 5 scenarios) is nearly as high as the intra-rater reliability (rho = 0.90–0.95). Taken together, these findings suggest that the VANS could be a “standardized” PN score across clinicians, not just a clinician-dependent score.

### Limitations

For our study, we attempted to oversample participants with PN-F to better define the cutoff score for PN. We had fewer participants with PN+F, and we would need a larger sample to confidently speak about the ability of the VANS to classify PN severity. Evaluating participants over time would also strengthen our conclusions about the reliability and validity of using VANS to longitudinally track disease progression.

Selecting a “gold standard” for PN presence and severity is challenging given known shortcomings of ancillary tests. For example, in our practice, we do not routinely perform autonomic tests or nerve biopsies to delineate small-fiber neuropathy. Moreover, the sensitivities of these specialized procedures are low (25). The variable completion, timing, and method of nerve conduction studies (NCS) in this cohort made electrodiagnostics suboptimal for comparison to the VANS. Here, we more broadly recruited and classified previous neurology patients based on symptoms and signs of PN as is custom in clinical practice (7, 23). We believe this to be a fair approach. We chose to use the medical record-derived diagnosis and the expert scoring of in-person board-certified neurologists as our gold standards for concordance and reliability testing. We plan to systematically conduct nerve conduction studies in future investigations and compare those results to the scores on the VANS.

Finally, the VANS is the standard assessment of our tele-PN clinic in the GLA VA, but we do add additional examination tests to rule out PN mimics or further evaluate co-existing conditions when needed. As an example, we supervise more detailed pinprick testing of the hand and instruct provocative maneuvers in the case of carpal tunnel syndrome, or we add a straight leg raise for radiculopathy or quick leg raise for myelopathic tone. The VANS is intended to be a focused, remote PN exam in those patients for whom there is a significant pre-test probability of PN and does not replace a comprehensive neurological exam.

## Conclusions

The VA Neuropathy Scale (VANS) is a promising clinical assessment tool for diagnosing and monitoring the severity of PN in telemedicine settings. Rooted in existing PN instruments, the VANS has considerable face validity. This pilot study indicates early support for acceptable concordance and reliability of the VANS with in-person examinations. The VANS produces similar scores regardless of whether the exam is performed by a neurologist or technician or whether it is graded in-person or via telemedicine. We submit the VANS as a novel tool to augment neuromuscular evaluations via telemedicine.

## Data Availability Statement

The datasets for this study will not be made publicly available because the dataset was collected primarily for internal program evaluation/quality improvement purposes. The dataset is not authorized for secondary research at this time.

## Ethics Statement

This study was approved as a quality improvement activity by the Greater Los Angeles Veterans' Affairs Healthcare System (GLA VA) IRB.

## Author Contributions

All authors contributed to the design, data interpretation, and manuscript writing and revision of this research. AW and NJ contributed to the data collection. AW performed the data analysis and wrote the first version of the manuscript. All authors read and approved the submitted version.

### Conflict of Interest

The authors declare that the research was conducted in the absence of any commercial or financial relationships that could be construed as a potential conflict of interest.

## Abbreviations

AUC: area under the curve
CI: confidence interval
GLA VA: Greater Los Angeles Veterans' Affairs Healthcare System
ICC: intraclass correlation coefficient
MD: neurologist (medical doctor)
NCS: nerve conduction study
PN: polyneuropathy
ROC: receiver operating characteristic curve
TCT: telehealth care technician
TeleMD: tele-neurologist (medical doctor)
VANS: VA Neuropathy Scale.
